# Liquid Biopsy in the Clinical Management of High-Grade Serous Epithelial Ovarian Cancer—Current Use and Future Opportunities

**DOI:** 10.3390/cancers13102386

**Published:** 2021-05-14

**Authors:** Lara Paracchini, Maurizio D’Incalci, Sergio Marchini

**Affiliations:** 1 Department of Biomedical Sciences, Humanitas University, 20072 Pieve Emanuele, Italy; lara.paracchini@humanitasresearch.it; 2 IRCCS Humanitas Research Hospital, 20089 Rozzano, Italy; sergio.marchini@humanitasresearch.it; 3 Department of Oncology, Istituto di Ricerche Farmacologiche Mario Negri IRCCS, 20156 Milan, Italy

**Keywords:** high-grade serous epithelial ovarian cancer, liquid biopsy, circulating tumor DNA, highly sensitive technologies

## Abstract

**Simple Summary:**

As for other neoplasms, liquid biopsy can be a useful tool to improve diagnosis and to monitor the response to therapy of high-grade serous epithelial ovarian cancer, which is the most common and lethal gynecological malignancy. In this paper, we provide an overview of the available knowledge on the current status and future opportunities by the analysis of tumor-derived components circulating in the blood of high-grade serous epithelial ovarian cancer patients.

**Abstract:**

The lack of a sensitive and specific biomarker and the limits relating to the single primary tumor sampling make it difficult to monitor high-grade serous epithelial ovarian cancer (HGS-EOC) over time and to capture those alterations that are potentially useful in guiding clinical decisions. To overcome these issues, liquid biopsy has emerged as a very promising tool for HGS-EOC. The analysis of circulating tumor DNA appears to be feasible and studies assessing specific pathogenic mutations (i.e., *TP53*) or copy number alterations have shown a sufficient degree of sensitivity and specificity to be realistically used to monitor the effectiveness of antitumor therapy. Liquid biopsy can also provide potential important information on the mechanisms of sensitivity and resistance, e.g., by the determination of the reversion of *BRCA* mutations. Perspective studies are needed to test whether the application of liquid biopsy will significantly improve HGS-EOC management and patients’ survival.

## 1. Introduction

The possibility of developing a blood-based assay to survey the genomic landscape of human tumors and its dynamic evolution provides considerable clinical opportunities to optimize therapeutic regimens with the final aim of improving diagnosis and disease prognosis.

Exploring the genetic composition of tumors has been the major aim in cancer research for the last 20 years. Tumor genotyping has become a useful tool for clinical decisions, as it has the potential to identify, at the time of diagnosis, those patients who will likely respond to the intervention. The technological improvement made by the development and widespread use of high throughput sequencing technologies -i.e., Next Generation Sequencing, NGS- illuminated novel cancer-associated mutations at an unprecedented level and rate, making the hypothesis to profile the tumor genome of each patient realistic. For tumors, for which there is a clear evidence of a driving oncogene, the use of drugs design to act as specific inhibitors have provided clinically relevant therapeutic responses. This is the case, for example, for tumors harboring mutations in *PI3KCA, K-ras*, *B-raf*, *EGFR2* genes, or harboring gene amplification in the *ERBB2* gene or with *EML4–ALK* fusion gene, which are responsive to specific targeted inhibitors [1,2,3,4,5,6]. However, beyond these paradigmatic examples of successful target therapies, the impact of this approach into clinical practice has been below the expectation with limited improvements to patients’ care. In fact, as our knowledge about functional consequences of these genetic alterations rapidly grew, the implement of genomic analysis into routine clinical practice faced new issues and challenges—the most important of which is the dynamic changes that occur over time in the sub-clonal genome architecture of solid tumors, generally known as temporal heterogeneity [7].

To date, the tumor landscape analysis has required solid tumor biopsies as the main source of DNA material. However, tumor biopsies involve invasive procedures, which are often difficult to be repeated over time during and after the therapy, and is risky or even unfeasible in many cases. Based on the data reported in the literature, the idea that in most solid tumors it is now widely accepted, like epithelial ovarian cancer (EOC), that a single tissue biopsy analysis may present sample bias as it provides a tumor picture at a single location, within a single time point, thus missing spatial and temporal heterogeneity [8]. Gerlinger et al. observed for the first time tremendous spatial heterogeneity in multiple tumor suppressor genes [9]. This study highlighted the limits of single site tumor biopsy to achieve reliable biomarkers for treatment decisions and sheds new light on the molecular issues of primary resistance to chemotherapy. Moreover, considering relapsed disease after front line therapy, it has been shown that metachronous lesions do not necessarily mirror the biology of primary tumor, and thus they can acquire or lose genomic aberrations that are present in the primary lesions. For example, in a retrospective analysis of a small subset of EOC patients from whom matched synchronous and metachronous lesions, i.e., after chemotherapy treatment- were available, targeted re-sequencing analysis revealed that, almost 95% of somatic mutations were called as exclusive to each single lesion [10,11,12].

With this lack of knowledge about how potential therapy-related markers with sub-clonal origin may change throughout tumor progression, serial sampling for real time genomic-profiling becomes mandatory to provide crucial information to guide treatment decision and/or monitor treatment response. However, for the reasons described above, the disadvantages related the solid tumor biopsy limit the possibility to perform serial biopsies analysis in the clinical setting.

In conclusion, it is reasonable to suppose that the limited clinical advantage of most targeted therapies is due to the fact that treatment decisions are often made without any a priori knowledge of the correct genomic composition of the tumor at the time of treatment as they are largely based on the molecular picture taken through a single biopsy at diagnosis that cannot capture the evolution of tumor genomes over time and space.

Thus far, although primary tumor biopsy remains the gold standard to identify tumor associated genomic alterations, it is a clinical need to identify novel methods, especially in metastatic settings, for real time monitoring of the evolution of potentially prognostic and predictive biomarkers.

## 2. Liquid Biopsy: Potentially Useful Marker

For a long time, medicine has been permeated by the idea that the identification of diagnostic and prognostic biomarkers in biological fluids could help clinicians in early diagnosis and monitoring. Examples are the measurement in the bloodstream of different cancer-related biomarkers, i.e., alfa-fetoprotein (AFP), carcinoembryonic antigen (CEA), prostate specific antigen (PSA) and cancer antigen 125 (CA-125), for detection and monitoring of colorectal, prostate and ovarian malignancies, respectively [13]. Over the last years, oncology research has focused its attention on the identification of new circulating biomarkers with diagnostic, prognostic and predictive relevance. However, despite the great number of studies done, the majority of them have shown very limited reliability [14]. The introduction 10 years ago by Pantel and Alix-Panabieres of the concept of “liquid biopsy” as an evolution of the traditional “solid tumor biopsy”, generated new hopes for cancer patients’ management [15].

To date, liquid biopsy can be considered an umbrella term, as under this definition, different biochemical and biological analysis can be developed on different body fluids, i.e., bloodstream, urine, pleural effusion or cerebrospinal fluid, for different purposes such as early diagnosis, monitoring the minimal residual disease (MRD) or to follow tumor dynamics evolution [16,17,18]. The main advantages of liquid biopsies over single tumor samples are that they are non-invasive and cheap. The information obtained from the analysis of circulating tumor components, i.e., circulating tumor DNA (ctDNA) or circulating tumor cells (CTCs), may be able to help gain insights into the molecular and genetic features that characterize the tumor. This avoids potential misconceptions inherent in the results from single samples from specific anatomical sites. Furthermore the rapid turnover of the circulating tumor components, ranging from a few minutes to several days, allows a real-time longitudinal monitoring of tumor evolution and identification of changes in the tumor genomic landscape, which might help guide therapeutic decisions [19,20,21].

To date, there are only a handful of FDA-approved tests available for use in clinical settings, such as the Cobas^®^ EGFR Mutation Test V2 for treatment monitoring of NSCLC patients, the Epi proColon^®^ test for the detection of colorectal cancer and recently the Guardant360 CDx and FoundationOne Liquid CDx, which provides clinically relevant information in multiple solid tumors. Considering the CTC assessment, the only FDA-approved test to date is the CellSearch^®^ CTC, which has been approved for the enumeration of CTCs in metastatic breast, colorectal and prostate cancers.

**Figure 1:**
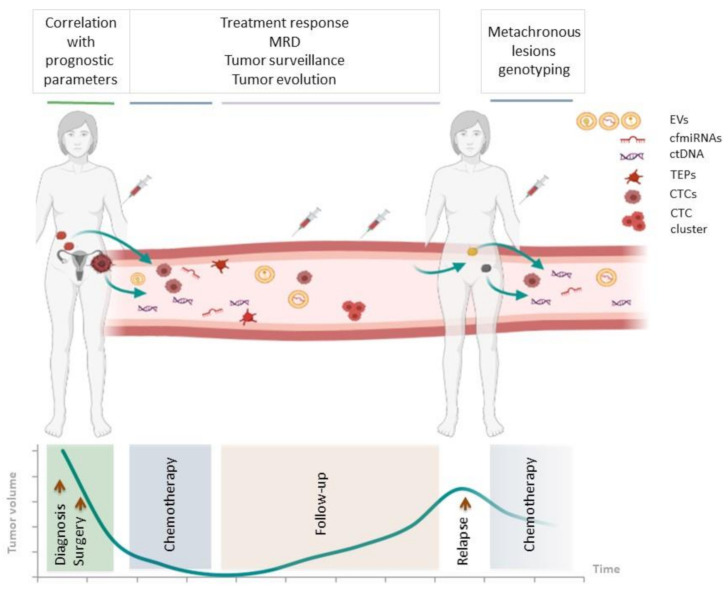
Applications of liquid biopsy in HGS-EOC. Scheme illustrating the information that can be obtained from the analysis of tumor-derived elements circulating in the bloodstream of HGS-EOC patients at different times, i.e., at the time of diagnosis, during and after chemotherapy, at follow up and relapse. MRD, minimal residual disease; EVs, extracellular vesicles; cfmiRNA, circulating-free microRNA; ctDNA, circulating-tumor DNA; TEP, tumor-educated platelet; CTC, circulating tumor cell.

Bloodstream analysis nowadays represents the most studied biological fluid for dynamic tumor genomic profiling and it will be the focus of the current review. In the bloodstream, it is possible to isolate different tumor-associated components, i.e., CTCs, also present as a cluster of cells, ctDNA, circulating extracellular vesicles (EVs) mainly exosomes, tumor-educated platelets (TEPs) and circulating-free microRNAs (cfmiRNAs) [22,23,24]. As highlighted in Figure 1, there are multiple potential applications of liquid biopsy. The same figure shows that the term of liquid biopsy includes many of tumor-related elements that can be investigated (Figure 1). Among the circulating tumor components, ctDNA is probably the most promising. Research done in the PubMed (NCBI) database typing “circulating-tumor DNA” as a search term evidences a nine-fold increase in ctDNA publications from 2011 to 2020, underlying the notable and growing interest of the scientific community in this particular field of research. The possibility to apply high-throughput technologies to circulating tumor-derived components has opened new hopes to investigate many solid tumors, although the lack of standardized or widely accepted procedures makes the application of liquid biopsy in clinical practice still limited.

Robust research is needed to enable the use of liquid biopsy in clinical practice, in particular for those solid tumors, such as high grade serous epithelial ovarian cancer (HGS-EOC), characterized by a strong spatial and temporal heterogeneity and for whom no up-to date reliable biomarkers are available yet.

In this paper, we have overviewed the recent advances of liquid biopsy application in HGS-EOC with particular attention to the recent improvements in the detection of cancer-related aberrations in circulating cell-free DNA (cfDNA) present in the plasma of HGS-EOC patients.

## 3. Liquid Biopsy in Ovarian Cancer

HGS-EOC is the most common and deadly subtype of EOC. It is a systemic and complex disease, with marked intra-tumor and inter-patients spatial and temporal heterogeneity [10,11,25]. The lack of both sensitive and accurate biomarkers for early diagnosis, and post-treatment surveillance, as well as the absence of molecular information of the disease at the recurrence might contribute to the high mortality rate of HGS-EOC.

Like colorectal, liver and breast cancers, the marked heterogeneity of HGS-EOC makes the use of a single tumor sampling analysis limited and inappropriate to correctly recapitulate the tumor genome landscape and its dynamic evolution [26,27,28,29]. For example, due to early pathogenic mutations in the *TP53* gene, the tumor genome is largely unstable with a progressive increase in different kinds of structural aberrations. In this scenario, biological information provided by the single primary tumor biopsy at diagnosis, when the tumor is naive to chemotherapy, does not allow us to identify those sub-clonal molecular alterations, e.g.*,* reversion of somatic *BRCA* mutations, which may have been developed in the metachronous lesions, thus characterizing the genome of relapsed disease and its resistance to conventional drugs [30,31,32].

The serum protein Cancer Antigen 125 (CA-125)—a glycoprotein encoded by *MUC16* gene—represents the only biomarker available in the clinic to monitor treatment response and early detection of recurrence. CA-125 is normally released by normal epithelial cells in the bloodstream at low concentrations, but its level dramatically increases under non-physiological conditions. In fact, although CA-125 is routinely used in clinical practice as a HGS-EOC biomarker (CA-125 cut-off > 35 U/mL), it is well demonstrated that its sensitivity and specificity are poor as its concentration in the bloodstream can also increase in benign conditions, such as endometriosis or pelvic inflammatory disease, as well as in other malignant tumors—lung, breast and gastrointestinal cancers [33,34,35]. Additionally, the computed tomography (CT) scan, the most common imaging modality usually used for disease surveillance, lacks sensitivity and is often delayed in demonstrating the relapse because of the inability to detect small tumor masses [36].

In this scenario, the use of liquid biopsy represents an innovative tool to overlook these issues. High-throughput technologies exploited to analyze the mentioned tumor-related components that are released in the bloodstream could permit to: (i) have a complete genotyping of the entire tumor burden at the time of diagnosis; (ii) track clonal aberrations that characterize HGS-EOC such as *TP53* mutations or 8q24 and 8q23 amplifications; and (iii) highlight tumor dynamic changes that occurs under a selective therapy pressure and obtain accurate information about the biology of relapse in order to guide the therapeutic decision [12,37].

In the following sections, studies performed so far on CTCs, EVs, and cfmiRNAs in HGS-EOC are reported, with particular attention to cfDNA that is currently considere the most promising and investigated circulating tumor derived component (Table 1).

## 4. CTCs, EVs and cfmiRNAs in HGS-EOC

HGS-EOC is a systemic disease with multiple synchronous and metachronous lesions geographically disseminated in multiple anatomical sites [62]. The metastatic dissemination can occur through two different, not yet characterized mechanisms: (i) the passive dissemination of tumor spheroid cells into the abdominal cavity by ascites; (ii) the release into the bloodstream of CTCs that are able to colonize other anatomical sites, such as the omentum, which is the preferred one. This model is in line with the “seed-and soil” hypothesis [63]. While blood vessels do not represent the preferential mechanisms of ovarian cancer tumor cells dissemination, blood draw represents a non-invasive, reproducible and low-risk tool to isolate and study tumor-derived components. Data collected over the last years suggest that blood-based analysis of tumor derived components is feasible and allows us to track molecular features of EOC evolution.

Since 2002, when Marth and colleagues first isolated CTCs in the peripheral blood of EOC patients, the interest of scientific community in investigating the predictive and prognostic role of CTCs is increased, although with inconclusive results [38]. In the first study, the authors exploiting immunobeads coated with MOC-31 antibody, isolated CTCs from the peripheral blood of 11 out of 90 (12%) EOC patients. This low detection rate could be explained, in part, by technical limitations and by the low sensitivity of the methodologies used for CTCs isolation and enrichment. Recently, the technological improvements and the use of both epithelial and mesenchymal markers for CTCs isolation increased the detection rate over the original 12% [41,48,64]. Multivariate analysis reported in the same paper did not show any statistically significant correlation between the presence of CTCs in the bloodstream before surgery and survival parameters like PFS or OS (*p*-value > 0.05). This result was further confirmed by Judson and colleagues (PFS; *p* = 0.72 and OS; *p* = 0.96) [39]. Many studies report investigations on the number of CTCs as a potential prognostic factor in EOC patients, but the overall results do not allow us to draw any firm conclusion. In particular, multiple studies highlighted the prognostic role of CTCs, showing that the amount of CTCs before surgery or after front line chemotherapy treatment was associated with poor prognosis in terms of both PFS and/or OS [40,41,42,43,44,45]. However, the results of these studies are not consistent as some further investigations failed to find similar correlations [46,47,48].

In addition to their prognostic significance, CTCs have also been investigated as potential biomarkers of chemotherapy responses in EOC. In two different studies, Obermayr and colleagues demonstrated that the number of CTCs, purified from bloodstream after chemotherapy, was significantly higher in non-responders compared to responders to platinum (pt)-based therapies (*p* = 0.0015 and *p* = 0.035, respectively)[42,44]. The development of CTCs analysis offered the opportunity to analyze, in real time, changes in the mRNA expression levels and their association with pt resistance. For example, Kuhlmann et al. in a cohort of 143 EOC patients demonstrated that high expression levels in CTCs of the excision repair cross-complementation group 1 (ERCC1), a gene involved in the resolution of DNA adducts induced by platinum compounds, was predictive of platinum resistance in both univariate (OR 5.79; 95% CI, 1.40–23.96; *p* = 0.027) and in multivariate (OR 8.5; 95% CI, 1.7–43.6; *p* = 0.010) analysis [49]. 

Several papers showed that in the late stages of the disease, the detection rate by the analysis of CTCs and CA-125 seems to be comparable. In contrast, in the early stages of the disease, the analysis of CTCs was a better predictor than CA-125 levels. Moreover, despite the limited number of studies and the small cohorts of patients, there is weak evidence that the analysis of CTCs could also outperform CA-125 in predicting the progression of the disease [45,48].

To date, data regarding the predictive and prognostic value of CTCs in EOC are often conflicting and do not allow us to consider CTCs as a robust biomarker that is useful to guide clinical decisions. While the analysis of CTCs is potentially useful to study tumor DNA, RNA and proteins and CTCs can be cultured to investigate the issue of drug resistance, some technical aspects still need to be implemented. In particular, the absence of a standardized methodology for CTCs isolation and enrichment, the low sensitivity of the techniques and the sampling bias of captured cells require a technical research effort before the introduction of CTCs in a clinical setting. EVs, which include microvesicles and exosomes, are membranous structures normally released into body fluids by most cells, including tumor cells [65]. EVs contain various bioactive molecules, such as proteins, lipids or nucleic acids, which are able to mediate inter-cellular communication, cell-ECM interactions and to induce microenvironment modifications promoting tumor growth, invasion and drug resistance [66,67,68]. The idea is that the cargo of biomolecules contained in the EVs makes these membranous structures representative of the cell of origin, constituting a sort of molecular fingerprint that can be used to monitor disease progression and response to therapy. With this aim, in the last years, the scientific community has explored the possible role of EVs as a potential biomarker for EOC. In 2018, in a cohort of 106 EOC patients, Pan and colleagues identified in EVs a significant enrichment in miR-21, miR-100, miR-200-b, miR-320 and a downregulation of other miRNAs, such as miR-16, miR-93, miR-126, miR-223, in comparison to healthy controls [50]. However, the low number of healthy cases (n = 29) and the lack of evaluation of the clinical significance of the combined 10 miRNAs makes this study very preliminary. In the same year, Yoshimura et al. from sera of 62 EOC patients identified the overexpression of miR-99a-5p in EVs compared to healthy women [69].

Not only miRNAs, but also EVs-associated proteins, suggest a possible application of EVs in the clinical management of EOC. For example, in 2019, Zhang et al. showed the role of four exosomal proteins—Lypopolysaccharide Binding Protein (LPB), Fibrinogen Gamma Chain (FGG), Fibrinogen Alpha Chain (FGA) and Gelsolin (GSN)—as diagnostic biomarkers in 40 EOC patients versus 40 non-cancerous control women. The same study shows that FGG and LBP provide information about patients’ prognosis (FGG; HR 0.97 for OS and 0.77 for PFS, CI 95%. LBP; HR 0.81 for OS and 0.77 for PFS, CI 95%) [51]. Another interesting study by Schwich and colleagues, in 78 EOC patients and 30 healthy female controls demonstrated a seven-fold increase in HLA-G levels in plasma circulating exosomes of EOC patients (mean 14.3 ng/mL) compared to healthy controls (1.9 ng/mL) (*p* < 0.0001) [52].

EVs, carrying different kind of tumor-derived components, could represent a sort of all-in-one biomarker, from whom several biological information regarding both tumor features and tumor-microenvironment interactions can be obtained. However, the absence of a standardized approach for EVs isolation and the limited sample size of the available studies make it impossible to reach definitive conclusions and further validations in a larger cohort of patients are needed.

MicroRNAs (miRNAs) are small regulator molecules of 21–25 nucleotides whose dysregulation in their expression levels lead to different pathological conditions, including cancer [70]. As miRNA are released from both tumor and stromal cells, they have the advantage to recapitulate the dynamic cross-talk between tumor and its microenvironment, thus making them suitable candidate biomarkers. The encapsulation in EVs mentioned above represents one of the two ways through which miRNAs can circulate into the bloodstream, as they can also be released in a cell-free mode bound to specific RNA-binding proteins (cfmiRNAs). 

One of the first study by Resnick et al. in 2008 showed the potential role of eight miRNAs such as miR-21, miR-92, miR-93, miR-126, miR-29a, miR-155, miR-127 and miR-99b- isolated from serum in discriminating EOC patients from healthy controls (*p* < 0.01), thus proving the feasibility of using cfmiRNAs for EOC detection [53]. In 2017 Todeschini et al. in two independent cohorts of HGS-EOC patients (n = 168) and healthy controls (n = 65) demonstrated the clinical relevance of miR-1246 as prognostic biomarker for HGS-EOC (AUC = 0.89). In particular, using a novel approach for microarray data analysis and normalization, authors demonstrated a significant increase in the expression levels of miR-1246 in sera of HGS-EOC compared to healthy individuals with a sensitivity of 87%, a specificity of 77% and accuracy of 84% [54]. Many other studies highlight the key diagnostic and prognostic role of cfmiRNAs belonging to the miR-200 family, in particular miR-200a, miR-200b and miR-200c [71,72,73,74]. The elements of the miR-200c family are key regulators of the Epithelial to Mesenchymal Transition (EMT) process, which is well known to be involved in drug resistance and tumor progression in EOC [75]. The obtained results confirm the key biological role of this class of miRNAs and stimulate further research in larger cohorts of patients.

The great number of studies and the poor consistency of the results prompted Wang et al. to undertake a meta-analysis to estimate the accuracy of cfmiRNAs in detecting EOC. Considering 13 studies published in the literature since 2017, the authors evidenced a sensitivity of 89% and a specificity of 68%, thus suggesting a moderate diagnostic accuracy of cfmiRNAs to identify EOC. cfmiRNA signatures were found to have a higher potential diagnostic value than a single cfmiRNA biomarker to detect EOC [76].

While the diagnostic and prognostic role of cfmiRNAs is suggested, the overall results are not sufficiently robust for a clinical application. From a technical point of view, the different experimental procedures used for cfmiRNAs isolation, the non-standardized normalization process and data analysis and the low statistical power of many studies certainly have contributed to generate controversial results in this field of research. To overcome these limits and to generate robust and reproducible results, further research is needed.

## 5. Circulating-Free and Circulating Tumor DNA

Analysis of ctDNA is currently the most promising circulating biomarker with expected clinical utility in the near future. The development of more and more accurate and sensitive high-throughput sequencing technologies and the implementation of bioinformatics tools, have made it possible to detect tumor related aberrations in cfDNA related to the presence of the disease and to identify genetic alterations associated with drug response.

The discovery of cell free-DNA (cfDNA) in the plasma of both healthy and sick subjects was described at first by Mandel and Metais in 1948 [77]. Under physiological conditions, the amount of cfDNA in the blood can range considerably (from 1 to 100 ng/mL of plasma) and the amount can increase after physical exercise or during pregnancy [78,79,80,81,82]. Approximately four decades ago, it was described for the first time that cfDNA plasma levels also dramatically increase under pathological conditions such as acute trauma, stroke, end-stage renal failure and cancer [83,84,85,86,87,88].

The discovery in 1977 that cfDNA plasma levels raise in cancer patients and in particular that this increase is due to the amount of DNA released from tumor cells -ctDNA- has dramatically highlighted the interest in this field, bringing out cfDNA as a potential source of material to better understand tumor biology and its dynamic evolution [89,90].

In physiological conditions, the majority of cfDNA in the bloodstream is mainly derived from hematopoietic cells, i.e., white blood cells and erythrocyte progenitors [91]. In cancer patients’ bloodstream, the fraction of ctDNA generally represents a very small percentage, it is variable and can range from 0.1% to 89% [20,92]. This variability depends on different clinical, biological and anatomical factors, such as the cellular growth-rate, the stage of the disease, the tumor localization and proximity to blood vessels [93]. While the biological mechanisms by which ctDNA is released in the bloodstream are not fully clarified yet, the evidence suggests that apoptosis could be one of these. In physiologic conditions, the apoptotic leftovers are generally cleared by infiltrating phagocytes; however, with the increasing of tumor mass they cannot be efficiently removed causing their accumulation and consequent release in the bloodstream [17]. In support of this proposed mechanism of ctDNA release, there is evidence that ctDNA has a fragment length comparable to the classic apoptotic frangment length pattern, corresponding to 167 bp (range 145–180 bp) and its multiples [88,94]. Interestingly, analyzing cfDNA derived from cancer patients, Mouliere and colleagues evidenced a different size distribution in cfDNA versus ctDNA fragments’ length; in particular, they demonstrated an enrichment in fragments size < 150bp in mutant ctDNA (41%) versus non-mutant cfDNA (21%), thus demonstrating the highly fragmented profile of ctDNA in comparison with cfDNA [95].

Once in the bloodstream, cfDNA becomes the target of circulating enzymes such as DNAse I, which lead to a rapid cfDNA degradation and a subsequent elimination through the liver, spleen and kidney [96,97].

While, to our knowledge, no studies estimated the half-life of cfDNA/ctDNA in the bloodstream in a rigorous way, it is largely accepted that its half-life in circulation can vary approximately from 15 min to 2.5 h. Despite this short half-life, ctDNA can be detected in plasma because its release into the bloodstream is supposed to be a continuum event. This rapid cfDNA turnover, associated with the possibility to detect and analyze its tumor-derived content, has allowed to real-time monitor the disease, thus overcoming the static picture of the disease provided by the single solid biopsy [98]. Moreover, the analysis of ctDNA released in the bloodstream by lesions present in different anatomical regions allows us to have a complete view of the overall biological features that characterize the disease, thus overcoming the issue of spatial heterogeneity.

A large number of studies report the importance of real-time tracking ctDNA for early tumor diagnosis in order to detect minimal residual disease (MRD) after surgery or pharmacological therapy and to monitor tumor clonal evolution and emerging molecular mechanisms of drug resistance, underlying the great potential of this approach in various types of cancer, including HGS-EOC.

## 6. ctDNA Analysis: The State of the Art for Ovarian Cancer

**Figure 2:**
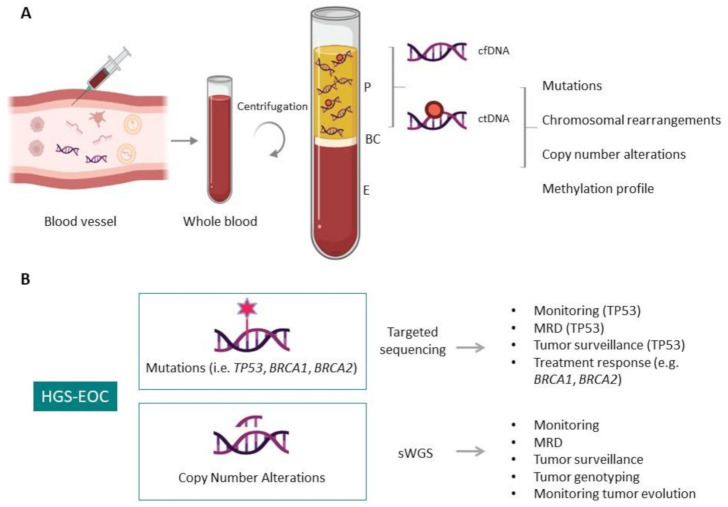
Circulating tumor DNA (ctDNA) in liquid biopsy. (**A**) Schematic representation of ctDNA isolation and genomic characteristic that distinguish ctDNA from cfDNA, providing information of the aberrations that characterize tumor masses. P, plasma; BC, buffy coat; E, erythrocytes. (**B**) Applications of ctDNA analysis in HGS-EOC. The information obtainable by target sequencing and shallow whole-genome sequencing (sWGS) are illustrated. MDR, minimal residual disease.

Initial studies evaluated the total cfDNA amount (Genome Equivalent, GE/ ml plasma) in patients with EOC in comparison to healthy controls and patients with benign ovarian tumors. An increase in the total amount of cfDNA was found in plasma of cancer patients. In addition, a significant difference between early and late stages of the disease was reported [93,99,100,101]. During the last years, the implementation of the next generation sequencing technologies and the development of new bioinformatic tools allowed us to improve the sensitivity of ctDNA analysis, despite the short ctDNA fragment length, making it feasible to monitor the most important genomic features of HGS-EOC, including the clonal pathogenic *TP53* mutation, other genes, e.g., *BRCA1* and *BRCA2,* involved in treatment response, and the chromosomal abnormalities that characterized HGS-EOC (Figure 2) [37,95,102,103,104].

To date, only a handful of observational studies focusing on the comparison between the CA-125/CT scan and ctDNA in predicting therapy response and anticipating disease recurrence in EOC are reported in the ClinicalTrials.gov website. Two phase 2 clinical trials are also registered. The first one, named CLIO trial (ClinicalTrials.gov Identifier: NCT02822157), is focused on the analysis of ctDNA to guide PARPi (Olaparib) treatment in recurrent epithelial carcinoma of the ovary, fallopian tubes, primary peritoneum cancers, while the second one (ClinicalTrials.gov Identifier: NCT04175470) has the goal to evaluate the variations of methylated HOXA9 ctDNA levels in pt-resistant EOC patients receiving bevacizumab. The results of these ongoing studies will be available, starting from 2023.

### 6.1. Somatic Mutation Detection

As a clonal driver pathogenic lesion, mutations in *TP53* gene are present in all tumor cells of the primary tumor mass, synchronous and metachronous lesions, thus representing a suitable biomarker to monitor the disease. The first studies that investigated the possibility to sequence cfDNA fragments to identify the presence of tumor-associated *TP53* mutation were published more than 15 years ago, and demonstrated the feasibility of this approach [55,56]. With the development of NGS technology, in 2016, Parkinson and colleagues used the clonal pathogenic *TP53* mutation identified in primary tumor samples to follow the mutant allelic fraction of the same mutation (TP53MAF) in matched longitudinal plasma samples by creating a digital PCR patients’ specific assay [57]. Applying this personalized approach, they first evidenced, in pre-treated HGS-EOC, a significant correlation between TP53MAF and tumor volume (Pearson r^2^ = 0.82), not evidenced for the standard HGS-EOC tumor biomarker CA-125 (Pearson r^2^ = 0.22). Interestingly in this work they demonstrated in multivariate analysis that a reduction > 60% in TP53MAF after the first cycle of front-line chemotherapy is associated with an increase in time to progression (TTP) of disease (HR 0.22, 95% CI 0.07–0.67, *p* = 0.008). On the contrary a reduction of <60% in TP53MAF was associated with a shorter TTP. Moreover, Parkinson et al. provided evidence of a faster dynamic increase of TP53MAF than CA-125 in predicting the progression of disease (median time to nadir 37 days IQR 28-54 for TP53MAF versus 84 days IQR 42-116 for CA-125). While with a limited number of patients, this study represents one of the first evidence of the great value of ctDNA analysis to monitor tumor burden and predict TTP compared favorably to the conventional CA-125 HGS-EOC tumor biomarker. Recently, Kim and colleagues have investigated the association between the mutant *TP53* allelic fraction identified in ctDNA collected after three months from the end of chemotherapy and prognostic parameters. Subdividing HGS-EOC patients on the basis of their mutant *TP53* allele count (TP53MAC) (cut-off: 0.2 copies/µL) they evidenced differences in TTP between the two groups (*p* = 0.038), thus confirming the significant prognostic utility of ctDNA compared to CA125 for HGS-EOC [58].

The analysis of *TP53* mutations in ctDNA appeared to be promising to evaluate tumor burden, to predict TTP and to monitor the disease overtime. However, *TP53* mutational analysis did not predict drug response thus suggesting the need of extending the analysis to other genes—e.g., *BRCA1* and *BRCA2—*whose mutations are associated with response to pt-compounds and PARP inhibitors (PARPi) [105,106].

The presence of germline or somatic pathogenic mutations in *BRCA* genes that are responsible for homologous recombination deficiency, is associated with increased response to treatment with DNA damaging agents and PARPi; however, the acquisition of new mutations that are able to restore the correct ORF is considered to be one of the various mechanisms of acquired chemotherapy resistance to PARPi. As a consequence, the tracking of *BRCA* mutations in ctDNA represents a possible strategy to monitor HGS-EOC during PARPi therapy [107,108]. In a recent study, Lin and colleagues have analyzed 112 HGS-EOC patients with germline or somatic *BRCA1\2* mutations enrolled in ARIEL2 trial (ClinicalTrials.gov Identifier: NCT01891344). cfDNA derived from plasma samples collected before PARPi (rucaparib) treatment and after disease progression was full-exons sequenced for *BRCA* genes to assess the presence of reverse *BRCA* mutations and evaluating their association with response to pt drugs and PARPi. They evidenced that, in pre-treatment cfDNA, the absence of reverse BRCA mutation was associated with both pt sensitivity (*p* = 0.049) and with a longer progression free-survival after rucaparib (HR 0.12; *p* < 0.0001). Moreover, in eight patients at the progression of the disease *BRCA* reverse mutations not present in pre-treatment plasma were identified, thus underlying the utility to follow the dynamic changes in *BRCA* mutational status to assess primary and acquired resistance to PARPi [59].

To sum up, targeted sequencing methods are undoubtedly promising, but they still suffer some technical limitations, including, but not limited to, the variable amount of cfDNA, artifacts introduced by PCR and the limit of detection in sequencing (approximately 0.4% of allelic fraction with 40 million reads per sample) [109]. Moreover, identification of low fraction variants requires very high coverage (>2000X, up to 20000X), leading high costs of analysis. Data reported in literature show a high level of variability between tumor-based and ctDNA-based mutations’ concordance, which could be explained by the nature of the mutations considered and by the fraction of ctDNA. Focusing on clonal mutations, the level of concordance is approximately 80%, but it decreases below 30% for sub-clonal mutations [57,107,110]. In addition, the limit of detection used to analyse ctDNA can impact on the concordance rate [110]. For these reasons, an untargeted whole-genome sequencing approach, able to capture the great somatic genomic alterations that characterizing HGS-EOC, using a low sequencing coverage, could represent a new cost-effective alternative to longitudinally track and monitor tumor evolution.

### 6.2. Structural Aberration Detection

The analysis of copy number alteration (CNA) profiling through a low-coverage whole-genome sequencing (0.2X–0.5X) has emerged both in solid tumor and in cfDNA as a suitable method to evaluate the chromosomal instability, thus allowing us to evaluate therapy response and dynamic tumor evolution, as well as to accurately quantify the tumor fraction in cfDNA without prior knowledge of solid tumor characteristics [111,112,113,114,115,116].

While this represents a promising approach, studies about its application on HGS-EOC are limited.

In 2017, Vanderstichele and colleagues demonstrated that the chromosomal instability quantification (genome-wide z-score) in cfDNA could be used to differentiate, at the time of diagnosis, patients with borderline or invasive carcinoma from those with benign adnexal masses. In particular, they showed the genome-wide CNA analysis on cfDNA outperforming in malignancy detection serum CA-125 and ultrasounds assessment [60].

A more extensive application of the low-pass whole-genome sequencing on cfDNA of HGS-EOC patients was proposed by Paracchini et al. who recently calculated the percentages of tumor fraction (TF) in cfDNA plasma samples collected at time of diagnosis and in longitudinal temporal windows and correlated them with clinical information. In particular, at time of diagnosis, TF was found to be an independent prognostic parameter (PFS HR= 3.31 CI 95% 1.33–9.13 *p* = 0.011). In longitudinal monitoring, Paracchini et al. demonstrated that the increase in TF outperformed CA-125 in predicting the disease progression. In particular, a significant increase in TF, arbitrary defined as an increase of >20% over TF baseline, was found to predict the radiological recurrence more accurately than CA-125 values, anticipating clinical and radiological progression with an average of eight months prior to its recurrence (range: 1–16). Interestingly, the tumor clonal evolution was tracked over time, showing the selective pressure induces by pt chemotherapy. The genomic heterogeneity of relapse disease was reduced after pt treatment, with the emergence of newly frequent cytobands that could be involved in t resistance. While this represents a proof-of-principle study performed in a small cohort of patients (n = 46) and lack of an independent clinical validation set, it provides evidence that the low-pass shallow whole-genome sequencing in cfDNA is an inexpensive and useful tool to monitor disease evolution and to anticipate relapse better than the routine clinical biomarkers [61].

The limitation of the low-pass whole genome sequencing approach, such as the lower sensitivity in detecting alterations involving <1 Mbp regions, is counterbalanced by its high-throughput nature, the speed of the analysis and the limited costs [104]. Moreover, considering the specific biological nature of HGS-EOC characterized by marked large chromosomal aberrations, it seems to be the most appropriate method to monitor and dissect the tumor evolution of the disease.

## 7. Conclusion and Future Directions

The development of liquid biopsy has been followed with great enthusiasm in the last decade. The development of a sensitive, specific and non-invasive approach to answer important clinical questions in terms of early diagnosis, prognosis, therapy response and disease monitoring has risen up great hopes, in particular for those cancers characterized by marked heterogeneity, such as the HGS-EOC. The present article overviews the main recent studies regarding the applications of liquid biopsy in HGS-EOC, and highlights the open questions that still remain to be answered. While some studies indicate that the approaches based on the measurement and characterization of CTCs, cfmiRNAs and EVs provide some important information on the biological features of EOC, the published results are not consistent enough to draw any firm conclusion on their practical clinical application. In our opinion, in the near future, the analysis of ctDNA seems to represent the most suitable and promising tool, particular for HGS-EOC. In fact, HGS-EOC is mainly characterized by two main genomic peculiarities: (i) the presence of an early clonal pathogenic mutation in the *TP53* gene, which is maintained both in space and over time; and (ii) the wide, genome scale, aspecific genomic structural alterations. In particular, two different technical approaches can be applied on cfDNA: the first one is a targeted approach focused on high deep analysis of a few driving lesions—*TP53* gene and other genes, e.g., *BRCA1* and *BRCA2*, which is helpful to guide PARPi therapy decisions—while the second one is represented by an untargeted approach such as sWGS, which is able to capture the great genomic aberrations, estimate the cfDNA TF and to capture the biological tumor evolution.

Data regarding the integration of the two approaches are still missing. It is reasonable to suppose that the longitudinal evaluation of MAFTP53, combined with the calculation of TF in cfDNA plasma samples, could further increase the sensitivity in detecting and longitudinal monitor HGS-EOC, thus increasing the potential of ctDNA analysis in anticipating disease recurrence in comparison to the current clinical biomarker CA-125. However, before applying liquid biopsy in clinical practice, it is important to set up precise guidelines to standardize all the pre-analytical and analytical variables in order to make the different platforms and the bioinformatics pipelines comparable for the correct variant calling. For example, the identification of mutations in *TP53* at very low allele frequency is very challenging due to various factors that impact the reliability of this analysis, including the low amount of cfDNA, as well as the presence of *TP53* mutations related to the physiological cellular turn-over or aberrant hematopoiesis processes. Besides these technical challenges, many other biological aspects that could impact on the final results need to be investigated with targeted studies. For example, precise studies aimed at evaluating the half-life and the clearance of cfDNA in the bloodstream of cancer patients, as well as the contribution in total cfDNA amount of the different metastasis, in relation to their localization and vascularization are required.

In conclusion, the use of liquid biopsy in an HGS-EOC setting appears to be feasible and useful. Therefore, it is time to insert this innovative approach in HGS-EOC clinical trials, and to test if its application will have a positive impact on the efficacy of treatment and HGS-EOC patients’ survival.

## Figures and Tables

**Table 1:** Applications of liquid biopsy in ovarian cancer studies. No. pt, number of patients; EOC, epithelial ovarian cancer; OS, overall survival; PFS, progression-free survival; NS, not significant; NA, not available; AC, after chemotherapy; BS, before surgery; CNA, copy number alteration.

CTCs Studies in Ovarian Cancer						
Author	No. pt	Subtype/Stage	Detection Rate	Prognostic Significance	Year	Ref
Marth et al.,	90	EOC (I-IV)	12% (BS)	NS	2002	[38]
Judson et al.,	53	EOC (I-IV)	19% (BS)	NS	2003	[39]
Aktas et al.,	122	EOC (I-IV)	19% (BS), 27% (AC)	OS (*p* = 0.005 BS and *p* = 0.004 AC). PFS, NS	2011	[40]
Poveda et al.,	216	EOC (I-IV)	CTCs ≥ 2 (12%) CTCs < 2 (88%) (BS)	OS (*p* = 0.0017) PFS (*p* = 0.00024)	2011	[41]
Obermayr et al.,	216	EOC (I-IV)	25% (BS)	OS (*p* = 0.001) PFS (*p* = 0.001) (AC)	2013	[42]
Chebouti et al.,	65	EOC (I-IV)	17% (BS)	OS (*p* = 0.0008) PFS (*p* = 0.0293) (AC)	2017	[43]
Obermayr et al.,	266	EOC (I-IV)	27%	OS (*p* = 0.007) PFS (*p* = 0.008) (AC)	2017	[44]
Zhang et al.,	109	EOC (I-IV)	90%	OS (*p* = 0.041) PFS, NS	2018	[45]
Kolostova et al.,	118	EOC (I-IV)	65%	NS	2015	[46]
Kolostova et al.,	56	EOC	58%	NS	2016	[47]
Guo et al.,	30	EOC (I-IV)	73%	NS	2018	[48]
Kuhlamann et al.,	143	EOC (I-IV)	14%	OS (*p* = 0.026) PFS (*p* = 0.009) (BS)	2014	[49]
**EVs and cfmiRNAs Studies in Ovarian Cancer**						
**Author**	**No. pt**	**Subtype/Stage**	**Biomarker**	**Prognostic Significance**	**Year**	**Ref**
Pan et al.,	106	EOC (I-IV)	miRNAs: miR-21, miR-100, miR-200b, miR-320,	NA	2018	[50]
			miR-16, miR-93, miR-126, miR-223			
Zhang et al.,	40	EOC (I-IV)	proteins: LBP, FGG, FGA, GSN	FGG: (OS *p* = 0.0012) (PFS *p* = 0.00038)	2019	[51]
				LBP: (OS *p* = 0.0029) (PFS *p* = 0.00023)		
Schwich et al.,	78	EOC (I-IV)	protein: HLA-G	PFS 3-years (*p* = 0.029) PFS 10-years (*p* = 0.006). OS, NS	2019	[52]
Resnick et al.,	28	EOC (I-IV)	miRNAs: miR-21, miR-92, miR-93, miR-126,	NA	2008	[53]
			miR-29a, miR-155, miR-127, miR-99b			
Todeschini et al.,	168	HGS-EOC	miRNA: miR-1246	NA	2017	[54]
**ctDNA Studies in Ovarian Cancer**						
**Author**	**No. pt**	**Subtype/Stage**	**Biomarker**	**Prognostic Significance**	**Year**	**Ref**
Swisher et al.,	137	EOC (I-IV)	*TP53*	OS (*p* = 0.02) PFS, NS	2005	[55]
Otsuka et al.,	27	EOC (I-IV)	*TP53*	NA	2004	[56]
Parkinson et al.,	40	HGS-EOC	*TP53*	PFS (*p* = 0.008)	2016	[57]
Kim et al.,	61	HGS-EOC	*TP53*	PFS (*p* = 0.008)	2019	[58]
Lin et al.,	112	HGS-EOC	*BRCA1/BRCA2*	Rucaparib PFS (*p* < 0.0001)	2019	[59]
Vanderstichele et al.,	68	Adnexal masses	CNA profiling	NA	2017	[60]
Paracchini et al.,	46	HGS-EOC	CNA profiling	PFS (*p* = 0.011)	2020	[61]

## References

[1] Schilsky R.L. (2014). Implementing personalized cancer care. Nat. Rev. Clin. Oncol..

[2] Roskoski R. (2019). Small molecule inhibitors targeting the EGFR/ErbB family of protein-tyrosine kinases in human cancers. Pharmacol. Res..

[3] Vasan N., Razavi P., Johnson J.L., Shao H., Shah H., Antoine A., Ladewig E., Gorelick A., Lin T.-Y., Toska E. (2019). Double PIK3CA mutations in cis increase oncogenicity and sensitivity to PI3Kα inhibitors. Science.

[4] Bar-Sagi D., Knelson E.H., Sequist L.V. (2020). A bright future for KRAS inhibitors. Nat. Rev. Cancer.

[5] Hamid O., Cowey C.L., Offner M., Faries M., Carvajal R.D. (2019). Efficacy, safety, and tolerability of approved combination BRAF and MEK inhibitor regimens for BRAF-mutant melanoma. Cancers.

[6] Solassol I., Pinguet F., Quantin X. (2019). FDA- and EMA-approved tyrosine kinase inhibitors in advanced EGFR-mutated non-small cell lung cancer: Safety, tolerability, plasma concentration monitoring, and management. Biomolecules.

[7] Meric-Bernstam F., Mills G.B. (2012). Overcoming implementation challenges of personalized cancer therapy. Nat. Rev. Clin. Oncol..

[8] Kleppe M., Levine R.L. (2014). Tumor heterogeneity confounds and illuminates: Assessing the implications. Nat. Med..

[9] Gerlinger M., Rowan A.J., Horswell S., Math M., Larkin J., Endesfelder D., Gronroos E., Martinez P., Matthews N., Stewart A. (2012). Intratumor heterogeneity and branched evolution revealed by multiregion sequencing. N. Engl. J. Med..

[10] Beltrame L., Di Marino M., Fruscio R., Calura E., Chapman B., Clivio L., Sina F., Mele C., Iatropoulos P., Grassi T. (2015). Profiling cancer gene mutations in longitudinal epithelial ovarian cancer biopsies by targeted next-generation sequencing: A Retrospective study. Ann. Oncol..

[11] Paracchini L., Mannarino L., Craparotta I., Romualdi C., Fruscio R., Grassi T., Fotia V., Caratti G., Perego P., Calura E. (2016). Regional and temporal heterogeneity of epithelial ovarian cancer tumor biopsies: Implications for therapeutic strategies. Oncotarget.

[12] Ballabio S., Craparotta I., Paracchini L., Mannarino L., Corso S., Pezzotta M.G., Vescio M., Fruscio R., Romualdi C., Dainese E. (2019). Multisite analysis of high-grade serous epithelial ovarian cancers identifies genomic regions of focal and recurrent copy number alteration in 3q26.2 and 8q24.3. Int. J. Cancer.

[13] Henry N.L., Hayes D.F. (2012). Cancer biomarkers. Mol. Oncol..

[14] Duffy M.J. (2013). Tumor markers in clinical practice: A review focusing on common solid cancers. Med. Princ. Pract..

[15] Pantel K., Alix-Panabières C. (2010). Circulating tumour cells in cancer patients: Challenges and perspectives. Trends Mol. Med..

[16] Pan W., Gu W., Nagpal S., Gephart M.H., Quake S.R. (2015). Brain tumor mutations detected in cerebral spinal fluid. Clin. Chem..

[17] Diaz L.A., Bardelli A. (2014). Liquid biopsies: Genotyping circulating tumor DNA. J. Clin. Oncol..

[18] Siravegna G., Marsoni S., Siena S., Bardelli A. (2017). Integrating liquid biopsies into the management of cancer. Nat. Rev. Clin. Oncol..

[19] Meng S., Tripathy D., Frenkel E.P., Shete S., Naftalis E.Z., Huth J.F., Beitsch P.D., Leitch M., Hoover S., Euhus D. (2004). Circulating tumor cells in patients with breast cancer dormancy. Clin. Cancer Res..

[20] Kustanovich A., Schwartz R., Peretz T., Grinshpun A. (2019). Life and death of circulating cell-free DNA. Cancer Biol. Ther..

[21] Wan J.C.M., Massie C., Garcia-Corbacho J., Mouliere F., Brenton J.D., Caldas C., Pacey S., Baird R., Rosenfeld N. (2017). Liquid biopsies come of age: Towards implementation of circulating tumour DNA. Nat. Rev. Cancer.

[22] Siravegna G., Mussolin B., Venesio T., Marsoni S., Seoane J., Dive C., Papadopoulos N., Kopetz S., Corcoran R., Siu L. (2019). How liquid biopsies can change clinical practice in oncology. Ann. Oncol..

[23] Mader S., Pantel K. (2017). Liquid biopsy: Current status and future perspectives. Oncol. Res. Treat..

[24] Marrugo-Ramírez J., Mir M., Samitier J. (2018). Blood-based cancer biomarkers in liquid biopsy: A promising non-invasive alternative to tissue biopsy. Int. J. Mol. Sci..

[25] Tan T.Z., Heong V., Ye J., Lim D., Low J., Choolani M., Scott C., Tan D.S.P., Huang R.Y.-J. (2019). Decoding transcriptomic intra-tumour heterogeneity to guide personalised medicine in ovarian cancer. J. Pathol..

[26] Parikh A.R., Leshchiner I., Elagina L., Goyal L., Levovitz C., Siravegna G., Livitz D., Rhrissorrakrai K., Martin E.E., Van Seventer E.E. (2019). Liquid versus tissue biopsy for detecting acquired resistance and tumor heterogeneity in gastrointestinal cancers. Nat. Med..

[27] Russo M., Siravegna G., Blaszkowsky L.S., Corti G., Crisafulli G., Ahronian L.G., Mussolin B., Kwak E.L., Buscarino M., Lazzari L. (2016). Tumor heterogeneity and lesion-specific response to targeted therapy in colorectal cancer. Cancer Discov..

[28] Losic B., Craig A.J., Villacorta-Martin C., Martins-Filho S.N., Akers N., Chen X., Ahsen M.E., von Felden J., Labgaa I., DʹAvola D. (2020). Intratumoral heterogeneity and clonal evolution in liver cancer. Nat. Commun..

[29] Song J.-L., Chen C., Yuan J.-P., Sun S.-R. (2016). Progress in the clinical detection of heterogeneity in breast cancer. Cancer Med..

[30] Miller R.E., Leary A., Scott C.L., Serra V., Lord C.J., Bowtell D., Chang D.K., Garsed D.W., Jonkers J., Ledermann J.A. (2020). ESMO recommendations on predictive biomarker testing for homologous recombination deficiency and PARP inhibitor benefit in ovarian cancer. Ann. Oncol..

[31] Tew W.P., Lacchetti C., Ellis A., Maxian K., Banerjee S., Bookman M., Jones M.B., Lee J.-M., Lheureux S., Liu J.F. (2020). PARP Inhibitors in the management of ovarian cancer: ASCO guideline. J. Clin. Oncol..

[32] Foo T., George A., Banerjee S. (2021). PARP inhibitors in ovarian cancer: An overview of the practice-changing trials. Genes Chromosomes Cancer.

[33] Buamah P. (2000). Benign conditions associated with raised serum CA-125 concentration. J. Surg. Oncol..

[34] Meyer T., Rustin G.J.S. (2000). Role of tumour markers in monitoring epithelial ovarian cancer. Br. J. Cancer.

[35] Sölétormos G., Duffy M.J., Abu Hassan S.O., Verheijen R.H.M., Tholander B., Bast R.C., Gaarenstroom K.N., Sturgeon C.M., Bonfrer J.M., Petersen P.H. (2016). clinical use of cancer biomarkers in epithelial ovarian cancer: Updated guidelines from the european group on tumor markers. Int. J. Gynecol. Cancer.

[36] Testa A.C., Di Legge A., Bonatti M., Manfredi R., Scambia G. (2016). Imaging techniques for evaluation of uterine myomas. Best Pract. Res. Clin. Obstet. Gynaecol..

[37] Ahmed A.A., Etemadmoghadam D., Temple J., Lynch A.G., Riad M., Sharma R., Stewart C., Fereday S., Caldas C., deFazio A. (2010). Driver mutations in TP53 are ubiquitous in high grade serous carcinoma of the ovary. J. Pathol..

[38] Marth C., Kisic J., Kaern J., Tropé C., Fodstad Ø. (2002). Circulating tumor cells in the peripheral blood and bone marrow of patients with ovarian carcinoma do not predict prognosis. Cancer.

[39] Judson P.L., Geller M.A., Bliss R.L., Boente M.P., Downs L.S., Argenta P.A., Carson L.F. (2003). Preoperative detection of peripherally circulating cancer cells and its prognostic significance in ovarian cancer. Gynecol. Oncol..

[40] Aktas B., Kasimir-Bauer S., Heubner M., Kimmig R., Wimberger P. (2011). Molecular profiling and prognostic relevance of circulating tumor cells in the blood of ovarian cancer patients at primary diagnosis and after platinum-based chemotherapy. Int. J. Gynecol. Cancer.

[41] Poveda A., Kaye S.B., McCormack R., Wang S., Parekh T., Ricci D., Lebedinsky C.A., Tercero J.C., Zintl P., Monk B.J. (2011). Circulating tumor cells predict progression free survival and overall survival in patients with relapsed/recurrent advanced ovarian cancer. Gynecol. Oncol..

[42] Obermayr E., Castillo-Tong D.C., Pils D., Speiser P., Braicu I., Van Gorp T., Mahner S., Sehouli J., Vergote I., Zeillinger R. (2013). Molecular characterization of circulating tumor cells in patients with ovarian cancer improves their prognostic significance—A study of the OVCAD consortium. Gynecol. Oncol..

[43] Chebouti I., Kuhlmann J.D., Buderath P., Weber S., Wimberger P., Bokeloh Y., Hauch S., Kimmig R., Kasimir-Bauer S. (2016). ERCC1-expressing circulating tumor cells as a potential diagnostic tool for monitoring response to platinum-based chemotherapy and for predicting post-therapeutic outcome of ovarian cancer. Oncotarget.

[44] Obermayr E., Bednarz-Knoll N., Orsetti B., Weier H.-U., Lambrechts S., Castillo-Tong D.C., Reinthaller A., Braicu E.I., Mahner S., Sehouli J. (2017). Circulating tumor cells: Potential markers of minimal residual disease in ovarian cancer? A study of the OVCAD consortium. Oncotarget.

[45] Zhang X., Li H., Yu X., Li S., Lei Z., Li C., Zhang Q., Han Q., Li Y., Zhang K. (2018). Analysis of circulating tumor cells in ovarian cancer and their clinical value as a biomarker. Cell. Physiol. Biochem..

[46] Kolostova K., Matkowski R., Jędryka M., Soter K., Cegan M., Pinkas M., Jakabova A., Pavlasek J., Spicka J., Bobek V. (2015). The added value of circulating tumor cells examination in ovarian cancer staging. Am. J. Cancer Res..

[47] Kolostova K., Pinkas M., Jakabova A., Pospisilova E., Svobodova P., Spicka J., Cegan M., Matkowski R., Bobek V. (2016). Molecular characterization of circulating tumor cells in ovarian cancer. Am. J. Cancer Res..

[48] Guo Y.-X., Neoh K.H., Chang X.-H., Sun Y., Cheng H.-Y., Ye X., Ma R.-Q., Han R.P.S., Cui H. (2018). Diagnostic value of HE4+ circulating tumor cells in patients with suspicious ovarian cancer. Oncotarget.

[49] Kuhlmann J.D., Wimberger P., Bankfalvi A., Keller T., Schöler S., Aktas B., Buderath P., Hauch S., Otterbach F., Kimmig R. (2014). ERCC1-positive circulating tumor cells in the blood of ovarian cancer patients as a predictive biomarker for platinum resistance. Clin. Chem..

[50] Pan C., Stevic I., Müller V., Ni Q., Oliveira-Ferrer L., Pantel K., Schwarzenbach H. (2018). Exosomal MicroRNAs as tumor markers in epithelial ovarian cancer. Mol. Oncol..

[51] Zhang W., Ou X., Wu X. (2019). Proteomics profiling of plasma exosomes in epithelial ovarian cancer: A potential role in the coagulation cascade, diagnosis and prognosis. Int. J. Oncol..

[52] Schwich E., Rebmann V., Horn P.A., Celik A.A., Bade-Döding C., Kimmig R., Kasimir-Bauer S., Buderath P. (2019). Vesicular-bound HLA-G as a predictive marker for disease progression in epithelial ovarian cancer. Cancers.

[53] Resnick K.E., Alder H., Hagan J.P., Richardson D.L., Croce C.M., Cohn D.E. (2009). The detection of differentially expressed micrornas from the serum of ovarian cancer patients using a novel real-time PCR platform. Gynecol. Oncol..

[54] Todeschini P., Salviato E., Paracchini L., Ferracin M., Petrillo M., Zanotti L., Tognon G., Gambino A., Calura E., Caratti G. (2017). Circulating MiRNA landscape identifies MiR-1246 as promising diagnostic biomarker in high-grade serous ovarian carcinoma: A validation across two independent cohorts. Cancer Lett..

[55] Swisher E.M., Wollan M., Mahtani S.M., Willner J.B., Garcia R., Goff B.A., King M.-C. (2005). Tumor-specific P53 sequences in blood and peritoneal fluid of women with epithelial ovarian cancer. Am. J. Obstet. Gynecol..

[56] Otsuka J., Okuda T., Sekizawa A., Amemiya S., Saito H., Okai T., Kushima M. (2004). Detection of P53 mutations in the plasma dna of patients with ovarian cancer. Int. J. Gynecol. Cancer.

[57] Parkinson C.A., Gale D., Piskorz A.M., Biggs H., Hodgkin C., Addley H., Freeman S., Moyle P., Sala E., Sayal K. (2016). Exploratory analysis of TP53 mutations in circulating tumour DNA as biomarkers of treatment response for patients with relapsed high-grade serous ovarian carcinoma: A retrospective study. PLoS Med..

[58] Kim Y.M., Lee S.W., Lee Y.J., Lee H.Y., Lee J.E., Choi E.K. (2019). Prospective study of the efficacy and utility of TP53 mutations in circulating tumor DNA as a non-invasive biomarker of treatment response monitoring in patients with high-grade serous ovarian carcinoma. J. Gynecol. Oncol..

[59] Lin K.K., Harrell M.I., Oza A.M., Oaknin A., Ray-Coquard I., Tinker A.V., Helman E., Radke M.R., Say C., Vo L.-T. (2019). BRCA reversion mutations in circulating tumor DNA predict primary and acquired resistance to the PARP inhibitor rucaparib in high-grade ovarian carcinoma. Cancer Discov..

[60] Vanderstichele A., Busschaert P., Smeets D., Landolfo C., Van Nieuwenhuysen E., Leunen K., Neven P., Amant F., Mahner S., Braicu E.I. (2017). Chromosomal instability in cell-free DNA as a highly specific biomarker for detection of ovarian cancer in women with adnexal masses. Clin. Cancer Res..

[61] Paracchini L., Beltrame L., Grassi T., Inglesi A., Fruscio R., Landoni F., Ippolito D., Marchette M.d., Paderno M., Adorni M. (2020). Genome-wide copy number alterations in circulating tumor dna as a novel biomarker in high grade serous ovarian cancer patients. Clin. Cancer Res..

[62] Lengyel E. (2010). Ovarian cancer development and metastasis. Am. J. Pathol..

[63] Yeung T.-L., Leung C.S., Yip K.-P., Yeung C.L.A., Wong S.T.C., Mok S.C. (2015). Cellular and molecular processes in ovarian cancer metastasis. A review in the theme: Cell and molecular processes in cancer metastasis. Am. J. Physiol. Cell. Physiol..

[64] Liu J.F., Kindelberger D., Doyle C., Lowe A., Barry W.T., Matulonis U.A. (2013). Predictive value of circulating tumor cells (CTCs) in newly-diagnosed and recurrent ovarian cancer patients. Gynecol. Oncol..

[65] Théry C., Gho Y.S., Quesenberry P. (2019). Journal of extracellular vesicles: The seven year itch!. J. Extracell. Vesicles.

[66] Van Niel G., D’Angelo G., Raposo G. (2018). Shedding light on the cell biology of extracellular vesicles. Nat. Rev. Mol. Cell. Biol..

[67] Cicero A.L., Stahl P.D., Raposo G. (2015). Extracellular vesicles shuffling intercellular messages: For good or for bad. Curr. Opin. Cell. Biol..

[68] Yáñez-Mó M., Siljander P.R.-M., Andreu Z., Zavec A.B., Borràs F.E., Buzas E.I., Buzas K., Casal E., Cappello F., Carvalho J. (2015). Biological properties of extracellular vesicles and their physiological functions. J. Extracell. Vesicles.

[69] Yoshimura A., Sawada K., Nakamura K., Kinose Y., Nakatsuka E., Kobayashi M., Miyamoto M., Ishida K., Matsumoto Y., Kodama M. (2018). Exosomal MiR-99a-5p Is Elevated in sera of ovarian cancer patients and promotes cancer cell invasion by increasing fibronectin and vitronectin expression in neighboring peritoneal mesothelial cells. BMC Cancer.

[70] He L., Hannon G.J. (2004). MicroRNAs: Small RNAs with a big role in gene regulation. Nat. Rev. Genet..

[71] Meng X., Müller V., Milde-Langosch K., Trillsch F., Pantel K., Schwarzenbach H. (2016). Diagnostic and prognostic relevance of circulating exosomal MiR-373, MiR-200a, MiR-200b and MiR-200c in patients with epithelial ovarian cancer. Oncotarget.

[72] Zuberi M., Mir R., Das J., Ahmad I., Javid J., Yadav P., Masroor M., Ahmad S., Ray P.C., Saxena A. (2015). Expression of serum MiR-200a, MiR-200b, and MiR-200c as candidate biomarkers in epithelial ovarian cancer and their association with clinicopathological features. Clin. Transl. Oncol..

[73] Gao Y.-C., Wu J. (2015). MicroRNA-200c and MicroRNA-141 as potential diagnostic and prognostic biomarkers for ovarian cancer. Tumor Biol..

[74] Halvorsen A.R., Kristensen G., Embleton A., Adusei C., Barretina-Ginesta M.P., Beale P., Helland Å. (2017). Evaluation of prognostic and predictive significance of circulating MicroRNAs in ovarian cancer patients. Dis. Mark..

[75] Teeuwssen M., Fodde R. (2019). Wnt signaling in ovarian cancer stemness, EMT, and therapy resistance. J. Clin. Med..

[76] Wang X., Kong D., Wang C., Ding X., Zhang L., Zhao M., Chen J., Xu X., Hu X., Yang J. (2019). Circulating MicroRNAs as novel potential diagnostic biomarkers for ovarian cancer: A systematic review and updated meta-analysis. J. Ovarian Res..

[77] Mandel P., Metais P. (1948). Nuclear Acids In Human Blood Plasma. C. R. Seances Soc. Biol. Fil..

[78] Mouliere F., Robert B., Peyrotte E.A., Del Rio M., Ychou M., Molina F., Gongora C., Thierry A.R. (2011). High fragmentation characterizes tumour-derived circulating DNA. PLoS ONE.

[79] Breitbach S., Sterzing B., Magallanes C., Tug S., Simon P. (2014). Direct measurement of cell-free DNA from serially collected capillary plasma during incremental exercise. J. Appl. Physiol..

[80] Beiter T., Fragasso A., Hudemann J., Niess A.M., Simon P. (2011). Short-term treadmill running as a model for studying cell-free DNA kinetics in vivo. Clin. Chem..

[81] Vittori L.N., Tarozzi A., Latessa P.M. (2019). Circulating cell-free DNA in physical activities. Methods Mol. Biol..

[82] Lo Y.M., Corbetta N., Chamberlain P.F., Rai V., Sargent I.L., Redman C.W., Wainscoat J.S. (1997). Presence of fetal DNA in maternal plasma and serum. Lancet.

[83] Filho E.M.R., Simon D., Ikuta N., Klovan C., Dannebrock F.A., de Oliveira C.O., Regner A. (2014). Elevated Cell-free plasma DNA level as an independent predictor of mortality in patients with severe traumatic brain injury. J. Neurotrauma.

[84] Ohayon S., Boyko M., Saad A., Douvdevani A., Gruenbaum B.F., Melamed I., Shapira Y., Teichberg V.I., Zlotnik A. (2012). Cell-free DNA as a marker for prediction of brain damage in traumatic brain injury in rats. J. Neurotrauma.

[85] Vajpeyee A., Wijatmiko T., Vajpeyee M., Taywade O., Pandey S., Chauhan P.S. (2020). Clinical usefulness of cell-free DNA as a prognostic marker in acute ischemic stroke. Neurologist.

[86] Tsai N.-W., Lin T.-K., Chen S.-D., Chang W.-N., Wang H.-C., Yang T.-M., Lin Y.-J., Jan C.-R., Huang C.-R., Liou C.-W. (2011). The value of serial plasma nuclear and mitochondrial DNA levels in patients with acute ischemic stroke. Clin. Chim. Acta.

[87] Einbinder Y., Shnaider A., Ghanayem K., Basok A., Rogachev B., Lior Y., Haviv Y.S., Cohen-Hagai K., Nacasch N., Rozenberg I. (2020). Elevated circulating cell-free DNA in hemodialysis-treated patients is associated with increased mortality. Am. J. Nephrol..

[88] Schwarzenbach H., Hoon D.S.B., Pantel K. (2011). Cell-free nucleic acids as biomarkers in cancer patients. Nat. Rev. Cancer.

[89] Leon S.A., Shapiro B., Sklaroff D.M., Yaros M.J. (1977). Free DNA in the serum of cancer patients and the effect of therapy. Cancer Res..

[90] Stroun M., Anker P., Maurice P., Lyautey J., Lederrey C., Beljanski M. (1989). Neoplastic characteristics of the DNA found in the plasma of cancer patients. Oncology.

[91] Moss J., Magenheim J., Neiman D., Zemmour H., Loyfer N., Korach A., Samet Y., Maoz M., Druid H., Arner P. (2018). Comprehensive human cell-type methylation atlas reveals origins of circulating cell-free dna in health and disease. Nat. Commun..

[92] Diehl F., Schmidt K., Choti M.A., Romans K., Goodman S., Li M., Thornton K., Agrawal N., Sokoll L., Szabo S.A. (2008). Circulating mutant DNA to assess tumor dynamics. Nat. Med..

[93] Bettegowda C., Sausen M., Leary R.J., Kinde I., Wang Y., Agrawal N., Bartlett B.R., Wang H., Luber B., Alani R.M. (2014). Detection of circulating tumor DNA in early- and late-stage human malignancies. Sci. Transl. Med..

[94] Sanchez C., Snyder M.W., Tanos R., Shendure J., Thierry A.R. (2018). New Insights into structural features and optimal detection of circulating tumor DNA determined by single-strand DNA analysis. NPJ Genom. Med..

[95] Mouliere F., Chandrananda D., Piskorz A.M., Moore E.K., Morris J., Ahlborn L.B., Mair R., Goranova T., Marass F., Heider K. (2018). Enhanced detection of circulating tumor DNA by fragment size analysis. Sci. Transl. Med..

[96] Lo Y.M., Zhang J., Leung T.N., Lau T.K., Chang A.M., Hjelm N.M. (1999). Rapid clearance of fetal DNA from maternal plasma. Am. J. Hum. Genet..

[97] Yu S.C.Y., Lee S.W.Y., Jiang P., Leung T.Y., Chan K.C.A., Chiu R.W.K., Lo Y.M.D. (2013). High-resolution profiling of fetal DNA clearance from maternal plasma by massively parallel sequencing. Clin. Chem..

[98] Elazezy M., Joosse S.A. (2018). Techniques of using circulating tumor DNA as a liquid biopsy component in cancer management. Comput. Struct. Biotechnol. J..

[99] Shao X., He Y., Ji M., Chen X., Qi J., Shi W., Hao T., Ju S. (2015). quantitative analysis of cell-free DNA in ovarian cancer. Oncol. Lett..

[100] Kamat A.A., Sood A.K., Dang D., Gershenson D.M., Simpson J.L., Bischoff F.Z. (2006). Quantification of total plasma cell-free DNA in ovarian cancer using real-time PCR. Ann. N. Y. Acad. Sci..

[101] Capizzi E., Gabusi E., Grigioni A.D., De Iaco P., Rosati M., Zamagni C., Fiorentino M. (2008). Quantification of free plasma DNA before and after chemotherapy in patients with advanced epithelial ovarian cancer. Diagn. Mol. Pathol..

[102] Dann R.B., DeLoia J.A., Timms K.M., Zorn K.K., Potter J., Flake D.D., Lanchbury J.S., Krivak T.C. (2012). BRCA1/2 mutations and expression: Response to platinum chemotherapy in patients with advanced stage epithelial ovarian cancer. Gynecol. Oncol..

[103] Pennington K.P., Walsh T., Harrell M.I., Lee M.K., Pennil C.C., Rendi M.H., Thornton A., Norquist B.M., Casadei S., Nord A.S. (2014). Germline and somatic mutations in homologous recombination genes predict platinum response and survival in ovarian, fallopian tube, and peritoneal carcinomas. Clin. Cancer Res..

[104] Adalsteinsson V.A., Ha G., Freeman S.S., Choudhury A.D., Stover D.G., Parsons H.A., Gydush G., Reed S.C., Rotem D., Rhoades J. (2017). Scalable whole-exome sequencing of cell-free DNA reveals high concordance with metastatic tumors. Nat. Commun..

[105] Patch A.-M., Christie E.L., Etemadmoghadam D., Garsed D.W., George J., Fereday S., Nones K., Cowin P., Alsop K., The Australian Ovarian Cancer Study Group (2015). Whole–Genome characterization of chemoresistant ovarian cancer. Nature.

[106] Vencken P.M.L.H., Kriege M., Hoogwerf D., Beugelink S., van der Burg M.E.L., Hooning M.J., Berns E.M., Jager A., Collée M., Burger C.W. (2011). Chemosensitivity and outcome of BRCA1- and BRCA2-associated ovarian cancer patients after first-line chemotherapy compared with sporadic ovarian cancer patients. Ann. Oncol..

[107] Weigelt B., Comino-Méndez I., de Bruijn I., Tian L., Meisel J.L., García-Murillas I., Fribbens C., Cutts R., Martelotto L.G., Ng C.K.Y. (2017). Diverse *BRCA1* and *BRCA2* reversion mutations in circulating cell-free DNA of therapy-resistant breast or ovarian cancer. Clin. Cancer Res..

[108] Ratajska M., Koczkowska M., Żuk M., Gorczyński A., Kuźniacka A., Stukan M., Biernat W., Limon J., Wasąg B. (2017). Detection of *BRCA1/2* mutations in circulating tumor DNA from patients with ovarian cancer. Oncotarget.

[109] Christensen E., Nordentoft I., Vang S., Birkenkamp-Demtröder K., Jensen J.B., Agerbæk M., Pedersen J.S., Dyrskjøt L. (2018). Optimized targeted sequencing of cell-free plasma DNA from bladder cancer patients. Sci. Rep..

[110] Bieg-Bourne C.C., Okamura R., Kurzrock R. (2020). Concordance between TP53 alterations in blood and tissue: Impact of time interval, biopsy site, cancer type and circulating tumor DNA burden. Mol. Oncol..

[111] Weiss G.J., Beck J., Braun D.P., Bornemann-Kolatzki K., Barilla H., Cubello R., Quan W., Sangal A., Khemka V., Waypa J. (2017). Tumor cell-free DNA copy number instability predicts therapeutic response to immunotherapy. Clin. Cancer Res..

[112] Van Roy N., Van Der Linden M., Menten B., Dheedene A., Vandeputte C., Van Dorpe J., Laureys G., Renard M., Sante T., Lammens T. (2017). shallow whole genome sequencing on circulating cell-free DNA allows reliable noninvasive copy-number profiling in neuroblastoma patients. Clin. Cancer Res..

[113] Davidson M., Barber L.J., Woolston A., Cafferkey C., Mansukhani S., Griffiths B., Moorcraft S.-Y., Rana I., Begum R., Assiotis I. (2019). Detecting and tracking circulating tumour DNA copy number profiles during first line chemotherapy in oesophagogastric adenocarcinoma. Cancers.

[114] Stover D.G., Parsons H.A., Ha G., Freeman S.S., Barry W.T., Guo H., Choudhury A.D., Gydush G., Reed S.C., Rhoades J. (2018). Association of cell-free DNA tumor fraction and somatic copy number alterations with survival in metastatic triple-negative breast cancer. J. Clin. Oncol..

[115] Heitzer E., Ulz P., Belic J., Gutschi S., Quehenberger F., Fischereder K., Benezeder T., Auer M., Pischler C., Mannweiler S. (2013). Tumor-associated copy number changes in the circulation of patients with prostate cancer identified through whole-genome sequencing. Genome. Med..

[116] Chen X., Chang C.-W., Spoerke J.M., Yoh K.E., Kapoor V., Baudo C., Aimi J., Yu M., Liang-Chu M.M.Y., Suttmann R. (2019). Low-pass whole-genome sequencing of circulating cell-free DNA Demonstrates dynamic changes in genomic copy number in a squamous lung cancer clinical cohort. Clin. Cancer Res..

